# Design of mini phantom and measurement of cobalt-60 beam data parameters

**DOI:** 10.4103/0971-6203.42750

**Published:** 2008

**Authors:** S. Senthilkumar, V. Ramakrishnan

**Affiliations:** Department of Radiotherapy, Govt. Rajaji Hospital and Madurai Medical Colege, Maduari-625 020, India; 1 Department of Laser Studies, Madurai Kamaraj University, Madurai, Tamil Nadu, India

**Keywords:** Collimator scatter correction factors (S_C_), collimator exchange effect, mini phantom, phantom scatter correction factors (S_P_), total scatter correction factors (S_C,P_)

## Abstract

Low cost mini phantoms were fabricated indigenously with different water equivalent material such as polymethyl methacrylate and Bee's wax of different shapes (with dome top surface and flat top surface). The beam parameters of the Co-60 machine, such as head scatter correction factor (S_h_), phantom scatter correction factor (S_P_), total scatter correction factor (S_C,P_), collimator exchange effect were measured. Output ratio measurements were taken for both mini phantom and water phantom for different square and rectangular field sizes. Normalized output ratios were compared with ESTRO published values and (Storchi and Van Gasteren) S and G data. The percentage of variation between the measured and the literature values is about 0.7%. Collimator exchange effect were measured for water and mini phantom for different field size, were compared with ESTRO value. This was found to be 0.5% and 1.0% respectively. Phantom scatter correction factors were calculated for square and rectangular filed sizes; this was compared with ESTRO values, found to be 0.7% for square and 1.0% for rectangular filed size. It was also noted that there were no appreciable variation observed in ion chamber readings of different materials of mini phantoms for dome and flat surfaces. Mini phantom measurements were done for all types of phantoms and the measured values were compared with the existing data and they were in good agreement with the published values.

## Introduction

In 2001 the ESTRO published the in-air measurements of head scatter components and volume scatter, output ratios, wedge factors and transmission tray factors etc. of high energy photon beam using mini phantom.[1] Mini-phantom play an important role for analysis of head scatter parameters.[2–4] The shape of the mini-phantom may be of square or circular cross section perpendicular to its long axis and it should be made of water equivalent materials such as polymethyl methacrylate (PMMA) or polystyrene.[5] The depth of the mini-phantom should be 10 cm to avoid the electron contamination and the diameter should be 4 cm to reach lateral electronic equilibrium.[6–8] The diameter of water volume, necessary to achieve quasi-lateral electron equilibrium increases slowly with increase of photon energy.[9] Measurements for output ratios, wedge factors and beam quality using polystyrene mini-phantom (relative electron density 1.02 g. cm^-3^) provides the quasi lateral equilibrium for high energy photon beam with a cross section of 4 × 4 cm^2^ mini phantom.[10,11]

The Netherlands commission on radiation dosimetry (NCRD) report states that the monitor unit calculation procedures were carried out by mini-phantom.[12] Normalized head scatter factor measurement and narrow beam coaxial mini-phantoms with build-up caps were used to conclude that the monitor unit calculation for the high energy photon beams affect the head scatter factor measurements due to the choice of the technique.[13] If build-up caps were made of metal, and the wall thickness is not enough or the build-up cap is not water equivalent, it may produce electron contamination. Many authors suggested that mini-phantom made by water equivalent material exclude the electron contamination and the output is directly proportional to the absorbed dose in water.[14]

Number of reports describes that the Head scatter (S_h_) measurement depends on the atomic number of the fabricating material of phantom.[15–18] Mini phantom material have no significant effect on Head scatter (S_h_) measurement.[19–21] All the above reports reveal that an effective atomic number close to that of water equivalent material is the best one for fabricating a mini phantom.

In this present work, a low cost and easy to handle in-house mini phantom has been fabricated, with two different water equivalent materials namely 1) PMMA (relative electron density 1.02), 2) Bee's wax, which are of two different shapes with top flat surface and dome. Phantom scatter correction factor for square and rectangular field size were calculated and these values were compared with the ESTRO published data and S and G values. Equal cross sectional dimension of 1 cm thickness PMMA material also were fabricated to analyze the characteristic of attenuation properties of mini phantom.

## Materials and Methods

Different types of build-up caps were used to measure the photon beam parameters; the basic columnar mini phantom is cylindrical with 4 cm in diameter and 25 cm in length. Mini phantoms were fabricated with different materials (PMMA and Bee's wax) with different surfaces like flat and dome. Dimension of the mini phantom and chamber holder are given in Table 1 and 2. These were kept in vertical direction and irradiated parallel to its long axis. The ion chamber was placed 10 cm below the surface of the mini phantom. When the photon beam travels through the long axis of the columnar mini phantom for a depth of 10 cm or so, it is deep enough to stop all the contaminating electrons in the provided buildup depth.

**Table 1:** Features and specification of in-house fabricated mini phantom

*Dimensions of phantom*	*PMMA phantom flat (cm)*	*PMMA phantom dome (cm)*	*Bees wax phantom flat (cm)*	*Bees wax phantom dome (cm)*	*PMMA slice (cm)*
Diameter	4	4	4	4	4
Length	25	25	25	25	1

PMMA - Polymethyl methacrylate

**Table 2:** Dimensions of mini phantom chamber holder

*Chamber Holder*	*Dimension (cm)*	*Base*	*Dimension (cm)*
Height	5	Thickness	2
Length	6	Length	30
Width	6	Width	30

Long axis of the mini phantom has the smallest cross section with respect to the photon beam and the ion chamber is placed along the cylindrical axis of the columnar mini phantom. The minimum field size of Co-60 machine is 5 × 5 cm^2^, so that the small size mini phantom will be irradiated fully leading to measure a correct head scatter.

**Figure 1:**
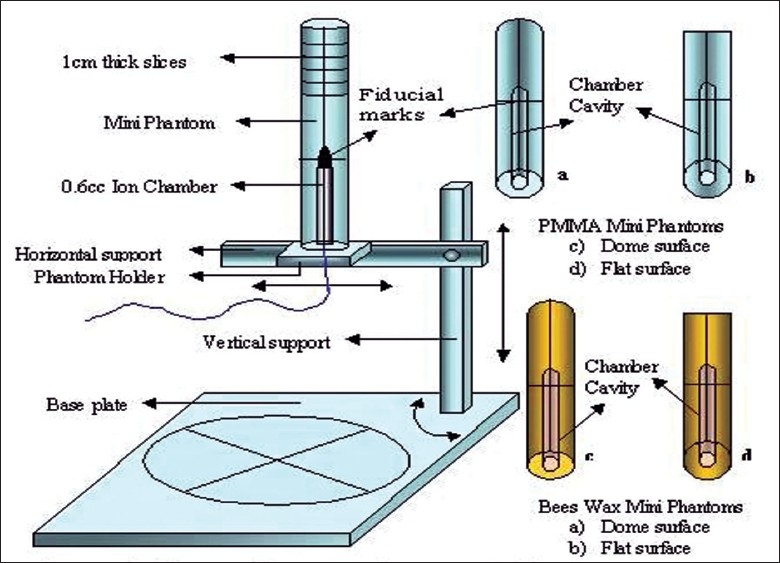
Block diagram of the PMMA and Bees wax mini Phantom and phantom holder setup with different types of mini phantom

**Figure 2:**
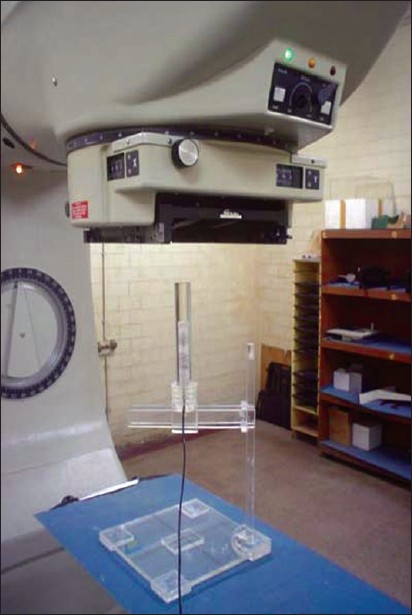
Photograph of mini phantom in the treatment position with Co-60 machine, which was fabricated for this study and used for dose measurement

Figure 1 shows the block diagram of the mini phantom experimental setup with different types of mini phantoms. Thimble 0.6cc cylindrical ion chamber was used as a detector and it was inserted in the mini phantom and held by a phantom holder. Figure 2 shows the mini phantom experimental setup in Co-60 machine. Mini phantom long axis was aligned parallel to the central axis of the photon beam with the help of laser alignment of the Co-60 room. The lines marked on the surface of the mini phantom can be used to position the central axis of the photon beam. The center of 0.6cc ion chamber active volume is set at a source-axis-distance (SAD) of 80 cm for Co-60 machine.

All the measurements were performed in the Co-60 Phoenix Theratron machine with average photon energy of 1.25MeV, which is equipped with symmetric collimator jaws. The dosimetry system used for this measurement is CD-high tech SSD Dosimeter and ion chamber used for this measurement is 0.6cc farmer type ion chamber Sl. No: CD-SSD-92/090. The minimum field size of the Co-60 machine is 5 × 5 cm^2^ and maximum field size is 35 × 35 cm^2^. The source to chamber distance (SCD) which is fixed for the dose measurement is equal to the Normal patient Treatment Distance (NTD) of Co-60 machine.[22] Before taking the measurement of the ion chamber, it was tested for stem effect and charge leakage test and was also irradiated to about 30 min. for chamber warm up.

### Output ratio measurements

**Figure 3:**
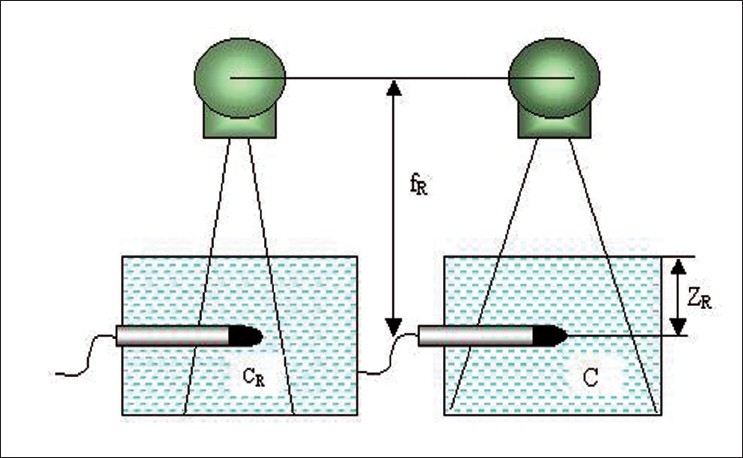
Experimental setup for output ratio O_R_(C) measurement in large water phantom for smaller and larger field size

**Figure 4:**
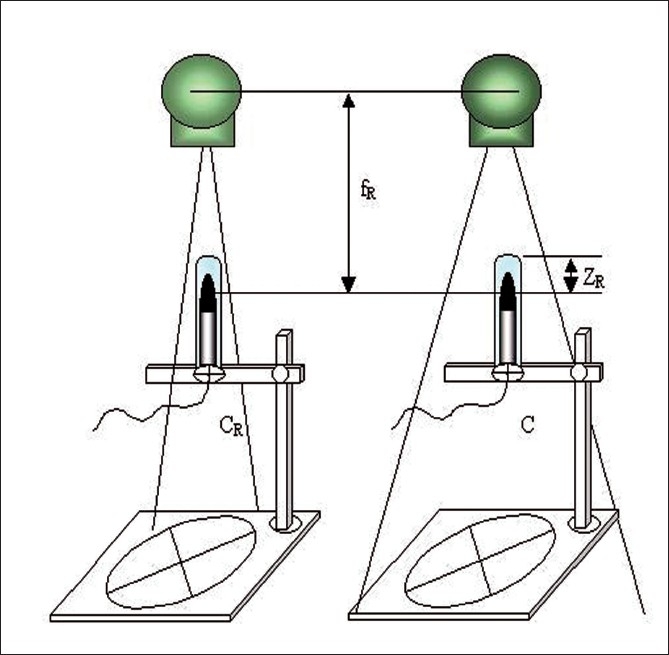
Experimental setup for output ratio O_O_(C) measurement in mini phantom for smaller and larger field size

a) Water phantom output ratio (O_R_) and mini phantom output ratio (O_O_) for square field measurement were carried out. Figure 3 shows the experimental setup for output ratio measurement in full scatter condition in a large water phantom of dimension 30 × 30 × 30 cm^3^. The 0.6cc ionization chamber was placed inside the water phantom at the reference depth of Z_R_ = 10 cm in the central axis of the photon beam. The source to chamber distance (SCD) was kept about 80 cm. Collimators X, Y were set for minimum equivalent square field of 5 × 5 cm^2^, exposed for a minute and meter readings were noted. Then the same procedure was repeated for all square and rectangular fields up to 35 × 35 cm^2^. A hole of diameter 1.5 cm and length 15 cm was made in the mini phantom to insert the ion chamber, which was used for O_O_ measurement [Figure 4]. Ion chamber is placed inside the phantom parallel to the central axis of the photon beam and the mini phantom was placed at the reference depth of 10 cm (Z_R_). The source to detector distance was kept about 80 cm. Collimators X and Y were set for minimum equivalent square field of 5 × 5 cm^2^, exposed for a minute and the meter readings were noted. Similar irradiating procedure of O_R_ was adopted for O_O_ measurement.

b) To measure O_R_ and O_O_ for rectangular field, the X collimator jaws were fixed at 5 cm and the Y jaws were moved from 5 cm to 35 cm. The ion chamber was exposed for a minute and the meter readings were noted. Similar procedure was repeated when the Y jaws was fixed at 5 cm and X jaws were moved from 5 cm to 35 cm.[23–25] Tatcher and Bjarngard also observed that the O_R_ and O_O_ is an asymmetric function of X and Y collimator settings.[26]

## Results and Discussion

Dose measurement in water phantom is essential to derive the output ratio under the full scatter condition. At the same time in-air measurement is also important. So we have used water phantom for output ratio measurement under the full scatter condition and mini phantom was used for in air measurement. Ionization chambers are the suitable one for the measurement of output ratio. Mini phantoms were used for in-air measurement of Co-60 output ratio (O_O_) for square field as well as for rectangular fields.[1] To get the accurate value of output ratio (O_O_), Collimator Exchange Effect (CEE) has to be included in the O_O_ calculation.

The basic method for separating scatter components of Co-60 machine, involves the measurement of the total scatter correction factor in a full phantom (S_C,P_) and the head scatter correction factor(S_h_).[27–31] The phantom scatter correction factor can be calculated as:

$$S_{p}=S_{\mathrm{c.p}}/S_{h}\to (1)$$

Where,

S_P_ - phantom scatter correction

S_C,P_ - full phantom scatter correction

S_h_ - head scatter correction

### Output ratio (O_R_) for square and rectangular fields in water phantom

Theratron phoenix Co-60 machine output ratio (O_R_) measurement has been carried out in a water phantom under the full scatter condition. Readings include head scatter and phantom scatter variations for all square field sizes(C) and the reference field size (C_R_). These values were compared with the ESTRO Booklet No: 6 published data for MDS Co-60 Theratron 780 values [Table 3]. TRS-398 and ESTRO Booklet No: 6 recommended that the output ratios O_R_(C) is the ratio of the absorbed dose at the reference depth for field size C, to the dose at the same depth for the reference field size C_R_, measured in a large water phantom, where both C and C_R_ are defined at the reference distances of 10 cm.[32]

**Table 3:** Output ratio (O_R_) for square fields in full scatter water phantom for Theratron Phoenix Co-60 compared with ESTRO Booklet No:6 published data for MDS Co-60 Theratron 780

*Side of square field (cm)*	*Theratron Phoenix Co-60*	*MDS Co-60 Theratron 780*	*% Deviation*
5	0.857	0.858	−0.12
6	0.887	0.897	−1.11
8	0.949	0.951	−0.21
10	1.000	1.000	0.00
15	1.081	1.093	−1.10
20	1.142	1.152	−0.87
25	1.183	1.200	−1.42
30	1.193	1.225	−2.61
35	1.204	1.236	−2.59

The output Ratio O_R_ in full scatter condition is,

$$O_{R}(C)=\frac{D(Z_{R},C)}{D(Z_{R},C_{R})}\to (2)$$

Where,

Z_R_ - reference depth,

C - the field size,

C_R_ - is the reference field size.

D - is the absorbed dose in the treatment condition at the reference distance Z_R_.

**Figure 5:**
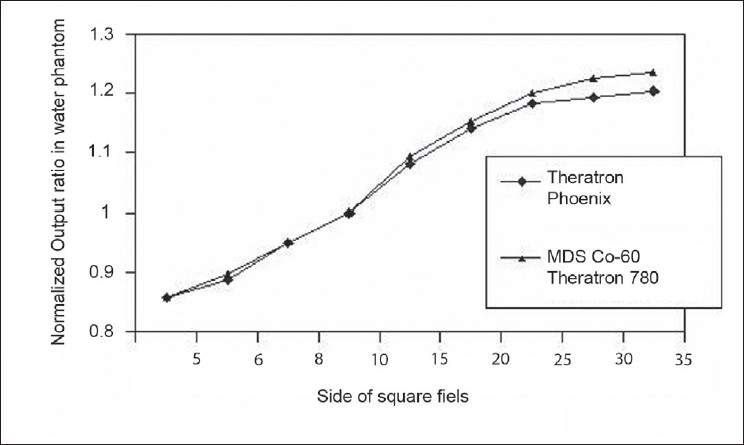
Output ratio (O_R_) in a Full scatter water phantom as a function of the side of a square field for Theratron Phoenix Co-60 compared with ESTRO Booklet 6 published data for MDS Co-60 Theratron 780

**Figure 6:**
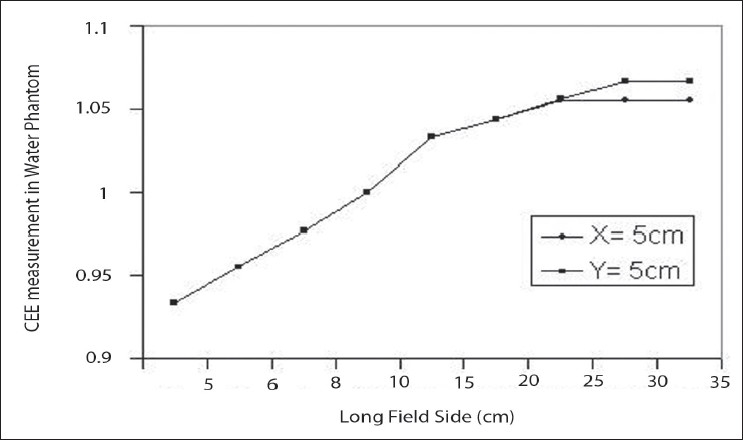
The Collimator Exchange Effect (CEE) for Theratron Phoenix Co-60 measured in a Water phantom

Figure 5 shows that both the values are almost same in the smaller square field and the output ratio increases with increase in field size. When the field size is increased water phantom scatter contribution also increases, resulting in a reduced in beam quality. Variation was observed in the larger filed size, indicating that the MDS Theratron 780 has more scatter contribution than the Theratron Phoenix machine. Table 4 Shows that the CEE of the Theratron phoenix machine values. The variation observed from the Figure 6 shows that the three curves are exhibiting the same trend, except for large field size. Collimator Exchange Effect is almost negligible in the Co-60 unit. This small variation is due to the collimator distance from the source, X jaws being nearer to the source than the Y jaws.

**Table 4:** Collimator exchange effect for Theratron Phoenix Co-60 measured with water phantom

*Long field size (X/Y)(cm)*	*Water phantom measurement O_R_(X,Y)*		*% Deviation*
	
	*x= 5 cm*	*Y= 5 cm*	
5	0.933	0.943	−1.06
6	0.955	0.955	0.00
8	0.977	0.988	−1.11
10	1.000	1.000	0.00
15	1.033	1.033	0.00
20	1.044	1.044	0.00
25	1.055	1.056	−0.09
30	1.0552	1.067	−1.11
35	1.055	1.1067	−1.12

## Output ratio (O_O_) for square and rectangular fields in mini phantoms

Theratron phoenix Co-60 machine output ratio O_O_ measurements were carried out in a mini phantom and the meter readings include mainly head scatter for a particular field size ‘C’ and the reference field size C_R_.

Output ratio (O_O_) in mini phantoms is:

$$O_{O}(C)=\frac{D_{O}(Z_{R},C)}{D_{O}(Z_{R},C_{R})}\to (3)$$

**Figure 7:**
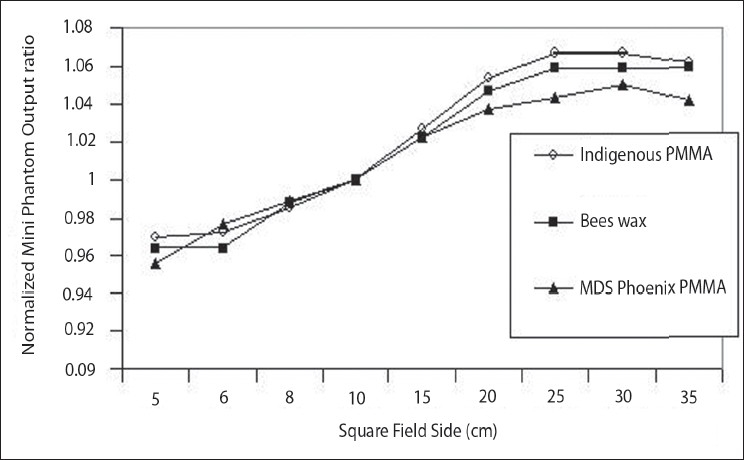
Output ratio (O_O_) in PMMA mini phantom and Bees Wax mini phantom readings were compared with MDS Co-60 phoenix PMMA mini phantom readings

**Figure 8:**
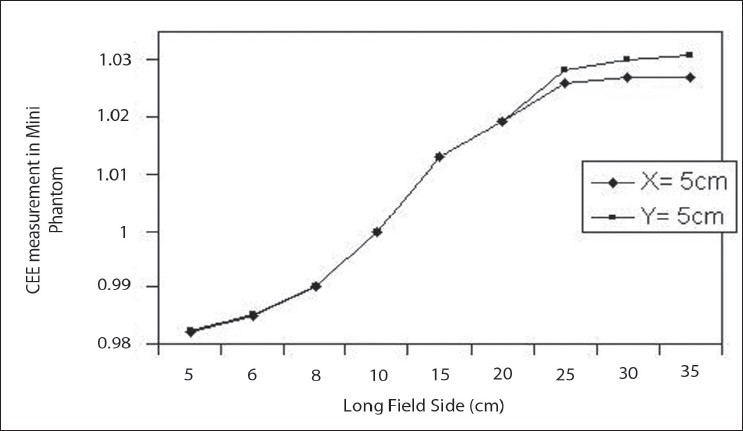
The Collimator Exchange Effect (CEE) for Theratron Phoenix Co-60 measured in a Mini phantom

Output ratio O_O_ values vary as the field size increases [Table 5]. Our mini phantoms values were slightly higher than the MDS Co-60 Theratron 780 values in the higher field size. Normalized output ratio of our mini phantoms values were compared with ESTRO Booklet 6 published data for MDS Co-60 Theratron 780 mini phantom values. Figure 7 shows that the three curves exhibit the same character. PMMA and Bee's Wax mini phantom gives the same output ratio with a small variation observed at higher filed size. The deviation between the two mini phantom values were also compared with the ESTRO Booklet 6 published data for MDS Co-60 Theratron 780 machine which was less than ± 0.5% in the both cases. Both O_O_ and O_R_ depend upon the orientation of the collimator for rectangular beams. Mini phantom measurement of CEE for the Theratron phoenix machine values are tabulated in Table 6 and these values are plotted in Figure 8. Both the X-Jaws and Y-Jaws curves were superimposed up to 20 × 20 cm^2^ field size. Some deviation is observed only in the larger field size similar to water phantom data. Thus the Theratron Phoenix Co-60 Machine has almost negligible Collimator exchange effect.

**Table 5:** Output ratio (O_o_) for square fields in PMMA mini phantom and Bees Wax mini phantom for Theratron Phoenix Co-60 compared with ESTRO Booklet 6 published data for MDS Co-60 Theratron 780

*Side of square field (cm)*	*Mini phantom measurements*		
	
	*Theratron Phoenix Co-60*		*MDS Co-60 Theratron 780*	*% Deviation*
	
	*PMMA*	*Bees wax*	*PMMA*	*PMMA*
5	0.970	0.964	0.955	1.57
6	0.973	0.964	0.977	−0.41
8	0.986	0.988	0.989	−0.30
10	1.000	1.000	1.000	0.00
15	1.027	1.023	1.023	0.39
20	1.054	1.047	1.038	1.54
25	1.067	1.059	1.044	2.20
30	1.067	1.059	1.051	1.52
35	1.062	1.059	1.042	1.92

PMMA - Polymethyl methacrylate

**Table 6:** Collimator exchange effect for Theraton Phoenix Co-60 measured with Mini phantom

*Long field size (X/Y)(cm)*	*Water phantom measurement O_o_(X,Y)*		% Deviation
		
	*X= 5 cm*	*Y= 5 cm*	
5	0.982	0.982	0.00
6	0.985	0.985	0.00
8	0.990	0.990	0.00
10	1.000	1.000	0.00
15	1.013	1.013	0.00
20	1.019	1.019	0.00
25	1.026	1.028	−0.19
30	1.027	1.030	−0.29
35	1.027	1.031	−0.39

## Total Scatter Correction Factor

### Phantom scatter correction factor for square field

Phantom scatter correction factor (S_P_) is a ratio of dose values between the full scatter condition in water phantom and in air measurement in a mini phantom.

$$i.e.S_{p}=\frac{O_{R}}{O_{O}}\to (4)$$

**Figure 9:**
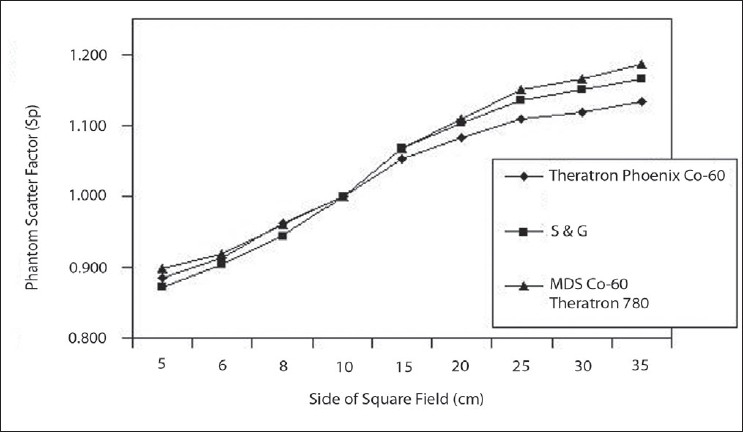
Comparison of Theratron Phoenix Co-60 Sp Values with the S and G values and ESTRO Booklet No:6 values for MDS Co-60 Theratron 780

Phantom scatter correction factor (S_P_) varies due to the beam quality with field size. S and G defined the phantom scatter correction factor for fixed SSD setup.[33] Calculated values are compared with S and G's ‘S_P_’ values and also with the ESTRO booklet No:6 values. Water phantom and mini phantom values were tabulated for all square field sizes and phantom scatter ratio results are presented in Table 7 and plotted in Figure 9 as a function of the square field size. These three curves are exhibiting the same, except at large field size. Our Co-60 values are similar to the other published datas. The result shows that the experimental S_P_ values lie ± 0.7% of the existing values.

**Table 7:** Phantom Scatter correction factor (Sp) of Theratron phoenix Co-60 for square fields compared with S and G data and MDS Co-60 Theratron 780 ESTRO Booklet No:6 published data

*Square field size (X/Y) (cm)*	*Theratron phoenix*	*S and G Data Co-60*	*MDS Co-60 Theratron 780*	*% Deviation with S and G data*
5	0.884	0.871	0.898	1.49
6	0.912	0.904	0.918	0.88
8	0.962	0.945	0.961	1.80
10	1.000	1.000	1.000	0.00
15	1.053	1.068	1.069	−1.40
20	1.083	1.104	1.110	−1.90
25	1.109	1.135	1.150	−2.29
30	1.118	1.150	1.166	−2.78
35	1.134	1.165	1.186	−2.66

PMMA - Polymethyl methacrylate

### Phantom scatter correction factor (S_P_) for rectangular fields

**Figure 10:**
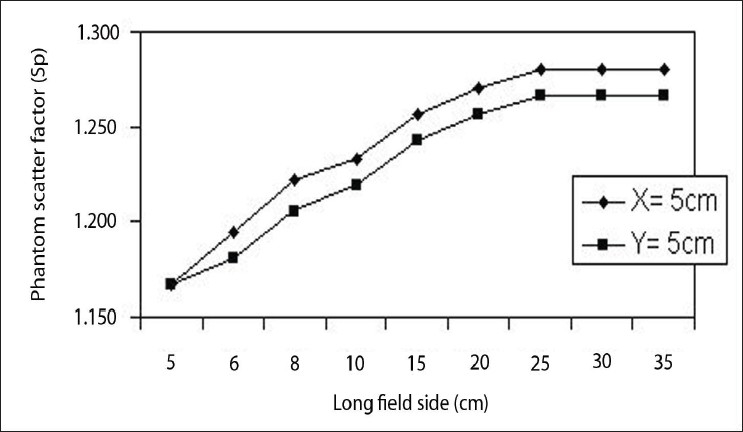
Phantom Scatter correction factor (Sp) for rectangular fields

The Phantom scatter correction factor (S_P_) for the dose contribution was derived from the water phantom scattered radiation and from the surrounding irradiated volume of the water phantom to the point of measurement on the central axis. Therefore S_P_ should be a symmetrical field size and S_P_ (X=a,Y=b) is equal to S_P_ (X=b, Y=a). This has been confirmed, within the experimental uncertainty, for all the high energy photon beams under investigation. Table 8 shows that the output ratio of O_R_ and O_O_ measurements for a given rectangular fields with independent setting of the X and Y jaws for the Co-60 beam. These values were directly obtained from the O_R_ and O_O_ measurements. From Figure 10 the deviation observed between measured and calculated values is about 1.0%. This variation might be due to the collimator distance from the radioactive source.

**Table 8:** Phantom Scatter correction factors (Sp) for rectangular fields

*Long field size (X/Y)(cm)*	*Phantom scatter correction factors S_P_ (X,Y)*	
	
	*X= 5 cm*	*Y= 5 cm*
5	1.167	1.167
6	1.194	1.181
8	1.222	1.205
10	1.233	1.219
15	1.257	1.243
20	1.270	1.257
25	1.280	1.267
30	1.280	1.267
35	1.280	1.267

### Comparison of flat and dome surface mini phantom

**Figure 11:**
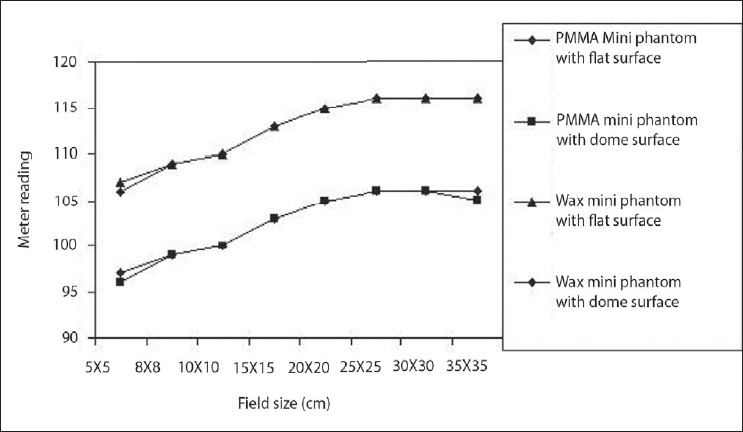
Comparison of the different shaped (flat and dome) PMMA and Bee's wax mini phantom as a function of field size at 80 cm SCD in Co-60 machine

Mini phantoms were fabricated for different surfaces – one is in flat and the other is dome shaped with different materials such as PMMA and Bee's Wax. All the four mini phantom output measurements were carried out (Source to Chamber Distance) SCD=80 cm for different field sizes. Electrometer readings are tabulated in Table 9 and they have been plotted in Figure 11. From the graph it was observed that some variation has been observed in the small field sizes and the larger field sizes. But there was no variation observed for values between these two extremes for both flat and dome shaped mini phantom of both the materials. The variation observed was very minimum and it is negligible for the change in the shape of surface.

**Table 9:** Comparisons of the different shapes PMMA and Bee's wax mini phantom output for different equivalent square field size

*Field size (cm)*	*PMMA mini phantom*			*Bees wax mini phantom*		
		
	*Flat surface*	*Dome surface*	*% Deviation*	*Flat surface*	*Dome surface*	*% Deviation*
5×5	97	96	1.04	107	106	0.94
8×8	99	99	0.00	109	109	0.00
10×10	100	100	0.00	110	110	0.00
15×15	103	103	0.00	113	113	0.00
20×20	105	105	0.00	115	115	0.00
25×25	106	106	0.00	116	116	0.00
30×30	106	106	0.00	116	116	0.00
35×35	106	105	0.95	116	116	0.00

PMMA - Polymethyl methacrylate

## Conclusion

The mini phantom fabricated using water equivalent material like PMMA and Bee's wax are comparatively easy to handle and it is of low cost. Water phantoms are generally used to measure the absolute dose to the point. This involves the primary and secondary components. In order to minimize the secondary components that arise from the water phantom, the irradiated volume was kept same. But it is not possible in the case of water phantom measurement. When increasing the field size for measurement, the irradiated volume also increases in larger field sizes. So, the phantom scatter contribution will be high. This type of water phantom has the disadvantage in measuring the head scatter factor. The diameter of the mini phantom is 4 cm and also has the large buildup cap. The irradiated volume is same for all field sizes. So it measures only the primary and collimator scatter of the secondary components and prevents the scatter contribution from the phantom. Mini phantom must have adequate wall thickness to stop the contaminating electrons from passing through the sides of the mini phantom to the chamber. Normalized output ratios were compared with ESTRO published values and S and G data. The percentage of variation between the measured and the literature values is about 0.7%. Collimator exchange effect were measured for water and mini phantom for different field size, were compared with ESTRO value. This was found to be 0.5% and 1.0% respectively. Phantom scatter correction factors were calculated for square and rectangular filed sizes; this was compared with ESTRO values, found to be 0.7% for square and 1.0% for rectangular filed size. It was also noted that there were no appreciable variation observed in ion chamber readings of different materials of mini phantoms for dome and flat surfaces. Mini phantom measurements were done for all types of phantoms and the measured values were compared with the existing data and they were in good agreement with the published values. This fabricated mini phantom can also be used for beam parameter measurement of Co-60 machine. The measurement of head scatter is independent of the orientation of the axis of the cylindrical ion chamber. Mini phantom dome surface and flat surface did not show any considerable change in the measurements. At the same time, the normalized value remains same for different water equivalent materials (PMMA and Bee's Wax). The results obtained were exactly same as that of previous literature review (Storchi and Van Gasteren and ESTRO Booklet No:6). Hence in-house fabricated mini phantom can also be used for Co-60 beam data parameter measurement.
